# Thy-1 Deficiency Augments Bone Loss in Obesity by Affecting Bone Formation and Resorption

**DOI:** 10.3389/fcell.2018.00127

**Published:** 2018-10-02

**Authors:** Ann-Kristin Picke, Graeme M. Campbell, Felix N. Schmidt, Björn Busse, Martina Rauner, Jan C. Simon, Ulf Anderegg, Lorenz C. Hofbauer, Anja Saalbach

**Affiliations:** ^1^Division of Endocrinology, Diabetes, and Bone Diseases, Department of Medicine III, Center for Healthy Aging, Technische Universität Dresden, Dresden, Germany; ^2^Institute of Comparative Molecular Endocrinology, Ulm University, Ulm, Germany; ^3^Institute of Biomechanics, TUHH Hamburg University of Technology, Hamburg, Germany; ^4^Department of Osteology and Biomechanics, University Medical Center, Hamburg, Germany; ^5^Department of Dermatology, Venerology, and Allergology, Medical Faculty, Leipzig University, Leipzig, Germany

**Keywords:** obesity, Thy-1, bone mass, osteoblast, osteoclast, adipocytes, differentiation, TNFα

## Abstract

Healthy bone remodeling results from a balanced bone formation and bone resorption realized by bone-forming osteoblasts and bone-resorbing osteoclasts, respectively. Recently, Thy-1 (CD90) was identified as positive regulator of osteoblast differentiation and activation, thus, promoting bone formation while concurrently inhibiting adipogenesis and obesity in mice. Additionally, Thy-1 did not affect bone resorption. An obesity-related co-morbidity that is increasing in prevalence is a disturbed bone formation resulting in an increased fracture risk. The underlying mechanisms of obesity-induced bone alterations are not yet fully elucidated and therefore therapy options for efficient bone-anabolic treatments are limited. Therefore, we investigated the impact of Thy-1 on bone metabolism under obese conditions. Indeed, high fat diet (HFD) induced obese mice lacking Thy-1 (Thy-1^−/−^) showed increased body fat mass compared to wildtype (WT) mice while bone mass (−38%) and formation (−57%) were decreased as shown by micro-computed tomography (μCT) measurement, histological analysis, and fourier-transform infrared spectroscopy (FTIR). Interestingly, under obese conditions, lack of Thy-1 affected both osteoblast and osteoclast function. Number (−30%) and activity of osteoblasts were decreased in obese Thy-1^−/−^ mice while osteoclast number (+39%) and activity were increased. Facilitated bone marrow fat accumulation (+56%) in obese Thy-1^−/−^ mice compared to obese WT mice was associated with upregulated tumor necrosis factor α (*Tnfα*, +46%) and colony stimulating factor 1 receptor *(Csf1r)* expression, strong promoters of osteoclast differentiation. Moreover, lack of Thy-1 was accompanied by a reduction of osteoprotegerin (*Tnfrsf11b*) expression (−36%), an inhibitor of osteoclast differentiation. Altered *Tnfα*, *Csf1r*, and *Tnfrsf11b* expression might be responsible for elevated osteoclast activity in obese Thy-1-deficient mice. In summary, our findings show that lack of Thy-1 promotes obesity under HFD conditions while concurrently decreasing bone mass and formation. Mechanistic studies revealed that under obese conditions lack of Thy-1 impairs both bone formation and bone resorption.

## Introduction

Healthy bone remodeling is a result of balanced bone formation realized bone-forming osteoblasts and bone-resorption mediated by bone resorbing osteoclasts (Crockett et al., 2011). Recently, Thy-1 (CD90) was identified as a critical molecule for the differentiation of osteoblasts and, thus, promoting osteogenesis and bone formation while inhibiting adipogenesis and obesity. Thy-1 is a glycosylphosphatidyl-anchored protein located on the cell surface of mesenchymal stem cells (MSCs), fibroblasts, activated microvascular endothelial cells, neurons, a subpopulation of hematopoietic stem cells (HSCs) and mouse T-cells (Vitetta et al., 1973; Craig, 1993; Wetzel et al., 2004; Schmidt et al., 2015; Picke et al., 2018a). Recently, it was discovered that Thy-1 controls fate decision of MSCs regarding differentiation into mature bone-forming osteoblasts or fat-storing adipocytes *in vitro* and *in vivo* (Hosoya et al., 2012; Chung et al., 2013; Woeller et al., 2015; Paine et al., 2018; Picke et al., 2018a). Mice lacking Thy-1 display a reduced osteogenic and increased adipogenic differentiation capacity, resulting in decreased bone mass and quality and concurrently, elevated body and bone marrow fat mass (Woeller et al., 2015; Paine et al., 2018; Picke et al., 2018a). Reduced osteogenesis in Thy-1 deficiency was linked to increased serum concentrations of the Wnt signaling inhibitors Dickkopf-1 (Dkk-1) and sclerostin, diminished Wnt ligand expression and attenuated Wnt signaling (Picke et al., 2018a). However, Thy-1 did not affect bone resorption in lean mice. The translational potential of these findings was underlined by the detection of strongly reduced levels of soluble Thy-1 in serum of patients with diminished bone formation such as in osteoporotic and obese patients (Picke et al., 2018a).

Worldwide, obesity is a major health problem negatively affecting bone metabolism. Obese patients often show increased bone mass and paradoxically suffer from an highly elevated fracture risk (Kling et al., 2014; Greco et al., 2015). Interestingly, in obesity bone remodeling is elevated in early phases due to increased mechanical loading by high body weight, which results in elevated bone mass (Greco et al., 2015). Later on, the massive amount of adipose tissue, especially bone marrow fat and visceral fat depots, leads to an increased production of pro-inflammatory cytokines such as tumor necrosis factor α (TNFα) and interleukin 6 (IL-6) resulting in chronic inflammatory response (Hotamisligil, 2006; Sharma et al., 2014; Palermo et al., 2016). This abnormal cytokine production results in altered bone mass and highly increased fracture risk in obese patients (Hsu et al., 2006; Nielson et al., 2012; Greco et al., 2015; Palermo et al., 2016).

Tumor necrosis factor α has the potential to increase the osteogenic differentiation capacity of MSCs or to reduce the osteogenic differentiation of pre-osteoblasts, which have already started their differentiation process (Gilbert et al., 2000; Osta et al., 2014). In addition, TNFα also promotes the differentiation of HSCs into osteoclasts by promoting actin ring formation and inducing an elevated secretion of receptor activator of NF-κB ligand (RANKL) by osteoblasts (Fuller et al., 2002; Osta et al., 2014). RANKL binds to its receptor RANK, located at the surface of osteoclast precursor cells, resulting in an increase in osteoclastogenesis. TNFα and RANKL have also been shown to operate synergistically on osteoclastogenesis by increasing RANK expression via TNF type 1 receptor (TNFR1) signaling (Zhang et al., 2001). In addition, CSF1, produced by osteoblasts, supports the positive effect of TNFα on osteoclastogenesis. Consequently, inhibition of CSF-1 in mice resulted in reduced osteoclastogenesis and osteolysis (Kitaura et al., 2005). Osteoblasts also produce osteoprotegerin (OPG), a decoy receptor of RANKL, and can therefore inhibit osteoclast differentiation (Boyce and Xing, 2008). In mice, high fat diet (HFD) reduces bone mass due to increased bone marrow adipogenesis and osteoclastogenesis mediated by higher levels of TNFα, RANKL, and PPARγ (Shu et al., 2015). The underlying mechanisms of HFD induced bone alterations are not yet fully elucidated and therefore, therapy options for efficient bone-anabolic treatments are limited (Tu et al., 2018).

In the present study, we analyzed the impact of Thy-1 on disturbed bone metabolism in obesity. Thy-1-deficient (Thy-1^−/−^) and wildtype (WT) mice were fed with a HFD to induce obesity. We detected a reduced number and activity of osteoblasts resulting in a decreased bone formation in obese Thy-1^−/−^ mice. In parallel, in obese Thy-1^−/−^ mice, osteoclast number and activity were increased. An elevated bone marrow adiposity associated with an increased pro-inflammatory environment including increased *TNFa* and *Csf1r* expression and an attenuated gene expression of OPG (*Tnfsf11B*), the decoy receptor for RANKL (*Tnfsf11*) in obese Thy-1^−/−^ mice contributed to strengthened bone resorption. Thus, under obese conditions, Thy-1 affects both the osteo-anabolic and -catabolic metabolism.

## Materials and Methods

### Mice

Thy-1 deficient (KO) mice on C57BL/6J background [kindly provided as a gift from Dr. R. Morris King’s College London, (61)] and C57BL/6J wildtype (WT) mice were kept under a 12-h light-dark cycle and given food and water *ad libitum*. All animal experiments were performed in accordance with institutional and state guidelines and approved by the Committee on Animal Welfare of Saxony (TVV 03/16, T26/16). Four to five weeks old male C57BL/6J mice were fed a HFD (EF R/M D12331 diet modified by Surwit, ssniff, Soest, Germany). The second cohort (**Supplementary Figure 1**; Picke et al., 2018a) were fed a standard chow until the age of 12 weeks.

### RNA Isolation, RT, and Quantitative Real Time PCR (qRT-PCR)

Total RNA from bone samples (ulnae) was isolated using a Trifast (PEQLAB, United States) method following the manufacturer’s instructions. For first strand cDNA synthesis with M-MLV reverse transcriptase (Promega, Madison, WI, United States), 1 μg total RNA was used according to the manufacturer’s protocol. Using *GoTaq^®^ qPCR Master* (Promega) the real-time qPCR was performed according to the manufacturer’s instructions on Rotor-Gene Q (QIAGEN). Used primers are listed in **Supplementary Table 1**. Quantitative gene expression was calculated from the standard curve of cloned cDNA and was normalized to the unregulated reference genes *Rs36* (*ex vivo* cell culture) *or Gapdh* (bones).

### Serum Analysis

Blood was drawn by heart puncture, centrifuged and frozen at −80°C. Serum levels of type 1 procollagen amino-terminal propeptide (P1NP), C-terminal telopeptide (CTX and P1NP: Immundiagnostik Systems, Germany), sclerostin (ALPCO, serum dilution 1:5), and Dkk-1 (R&D) were detected using immunoassay kits according to the manufacturer’s protocols.

### Assessment of Bone Mass, Microarchitecture, and Bone Marrow Fat Volume

Extracted bones were fixed for 48 h in 4% paraformaldehyde (PFA, Carl Roth, Karlsruhe, Germany) and were afterward dehydrated in 80% ethanol. By using the μCT vivaCT 40 (isotropic voxel size of 10.5 μm; 70 kVp, 114 μA, 200 ms integration time, Scanco Medical, Switzerland), femora and third lumbar vertebral bodies were analyzed as previously described (Picke et al., 2018a). The analysis of trabecular and cortical bone volume per total volume (BV/TV), bone mineral density (BMD), thickness (Tb.Th and Ct.Th for the trabecular and cortical thickness, respectively), trabecular number (Tb.N), trabecular separation (Tb.Sp) and cortical porosity (Ct.Po) was performed using established analysis protocols and the μCT parameters were reported according to international guidelines (63). To analyze the total area (Tt.Ar), marrow area (Ma.Ar) and cortical bone area (Ct.Ar) the periosteal and endosteal surfaces at the femoral mid-shaft were identified and afterward computed as the area within the periosteal boarder, the area within the endosteal border, and the area between the periosteal and endosteal borders, respectively (Picke et al., 2018a). Animations of the trabecular microstructure were generated from the Digital Imaging and Communications in Medicine (DICOM) image files in Amira (v6.0.0, Thermo Fisher Scientific, Hillsboro, OR, United States) and saved in gif format using ImageJ (v 1.46 r, NIH, United States; see **Supplementary Animation 1** of the trabecular bone compartment of femur of either WT or KO mice).

For assessment of the bone marrow fat content, fixed femora were decalcified (OSTEOSOFT^®^) for seven d and afterward scanned via μCT to ensure complete decalcification. They were then washed with PBS for 5 min, stained for 2 h with 2% osmium tetroxide (Electron Microscopy Science, United Kingdom) diluted in 0.1 M sodium cacodylate buffer (pH 7.4) (Picke et al., 2016), and were transferred into PBS. The complete femur was analyzed with μCT (70 kVp, 114 μA, 300 ms integration time, 10.5 μm isotropic voxel size) to evaluate the fat volume using the established protocols from Scanco Medical as previously reported e (Picke et al., 2018a).

### Fourier-Transform Infrared Spectroscopy (FTIR) Analysis

Fourier-transform infrared spectroscopy spectra was measured at a Spotlight 400 (PerkinElmer, Waltham, MA, United States) attached to a Frontier FTIR spectrometer (PerkinElmer, Waltham, MA, United States) in ATR-mode. Spectra were acquired within a wavelength range from 4000 to 570 cm^−1^ with a resolution of 4 cm^−1^. For each pixel (6.25 μm × 6.25 μm), 16 measurements were taken. SpectrumIMAGE software R.1.8.0.0410 (PerkinElmer, Waltham, MA, United States) was used for automatic background subtraction for each pixel spectrum and for automatic ATR correction. Each region of interest was 300 × 300 μm^2^ in size and included the whole cortical thickness. The spectra were post processed using a customized MATLAB (MATLAB 2014b, The MathWorks, Inc., MA, United States) routine with PMMA subtraction and baseline correction. Crystallinity was calculated by sub-peak fitting of the entire phosphate peak (1154–900 cm^−1^) and calculation of the ratio of the 1030 and 1020 cm^−1^ sub-peaks (27). The sub-peak of 1020 cm^−1^ is linked to non-stoichiometric apatite, whereas the sub-peak of 1030 cm^−1^ is linked to stoichiometric apatite (64). An increase of the crystallinity ratio reflects either an increase of stoichiometric apatite or a decrease of non-stoichiometric apatite (i.e., the crystallinity ratio reflects crystal size and perfection).

### Reference-Point Analysis, Three-Point Bending Test, and Femur Properties

The femora were shock frozen in liquid nitrogen after sacrifice and thawed from −80°C 5 min before reference-point indentation^TM^ (BioDent2, Active Life Scientific, United States). To avoid sample drying the bones were stabilized using an *ex vivo* small bone stage, which was filled with PBS. The reference probe was located at the anterior side of the femur shaft and indentation measurements were performed (2 N, five cycles) in triplicates for each bone sample by lifting up the measurement head unit and keeping the movement of the sample to a minimum. The total distance increase (TDI) was calculated. Promptly afterward, the femora were mechanically tested in a three-point bending (zwicki-Line 2.5 kN, Zwick, Germany). Load was applied at the anterior site of the femoral shaft until failure and the maximum load (F_max_, N) was recorded as previously reported (Picke et al., 2016, 2018a). The length (proximal to distal: greater trochanter to condyles) and width (shaft) of femora were measured using a caliper.

### Bone Histology and Histomorphometry

WT and Thy-1-deficient mice received i.p. injections of calcein (20 mg/kg) 5 and 2 days prior to sacrifice as previously reported (Picke et al., 2018a). The right and left proximal tibia were fixed in 4% PFA and dehydrated with 80% ethanol. For examination of bone formation rate by analysis of calcein labels (fluorescence), left tibiae were embedded in methyl methacrylate (Technovit 9100, Heraeus Kulzer, GER) and cut into 7-μm sections. BV/TV, bone formation rate per bone surface (BFR/BS), mineralized surface per BS (MS/BS), and mineral apposition rate (MAR) were determined in the trabecular part of the bone using the OsteoMeasure^®^ Software (OsteoMetrics, Atlanta, GA, United States) following the international standards (Parfitt et al., 1987). Mineralized bone areas (BV/TV) and osteoid surface per bone perimeter (Osteoid.S/B.Pm) were visualized by *von Kossa* staining and *van Gieson* counter staining. Therefore, bone sections were rehydrated using decreasing alcohol concentrations and were then sequentially exposed to silver nitrate (Roth), sodium carbonate (Merck), and sodium thiosulfate (Roth). Afterward, the slides were stained with toluidine blue (Waldeck GmbH, Germany) and dehydrated. The right proximal tibiae were then decalcified using OSTEOSOFT^®^ and embedded in paraffin (Leica Biosystems, United States) for the analysis of osteoclast numbers per bone surface (Oc.N/BS) and osteoclast surface per bone perimeter (Oc.S/B.Pm). Bone slices of 2 μm were stained at 37°C with tartrate resistant acid phosphatase (TRAP) staining solution (Naphthol-AS-BI-Phosphate, Fast Red Violet LB Salt, Triton X-100, dimethylformamide) and hemalum (Picke et al., 2018a).

### *Ex vivo* Experiments

Mesenchymal stem cells were isolated from femora and tibiae of Thy-1-deficient and WT mice by enzymatic digestion using 26 U/ml of Liberase DL (Roche) for 2 h at 37°C and 5% CO_2_. Cell suspension was filtered through a cell strainer (70 μm) and cultured in a-MEM medium (Lonza) supplemented with 10% fetal calf serum (FCS) (Thermo Fisher Scientific) and 1% penicillin/streptomycin (Biochrom AG) at 37°C, 5% CO_2_. CD11b-positive cells were removed by magnetic cell separation using anti-CD11b magnetic beads according to the manufacturer’s protocol (Miltenyi). Purity was checked as described previously (Picke et al., 2018a). MSCs were stimulated with 10 ng/ml TNF (Miltenyi) for 24 h.

### Statistical Analysis

The results are presented as mean ± standard deviation (SD). Distribution of data was assessed by Shapiro-Wilk test. Depending on the normality of the data, analysis was performed using the Mann-Whitney rank-sum test or the *t*-test. For assessment of the effect of Thy-1 deficiency and TNFα treatment on MSCs *ex vivo*, we performed two-way ANOVA with Tukey *post hoc* test. *P* < 0.05 was considered significant.

## Results

### Obese Thy-1^−/−^ Mice Display Decreased Trabecular Bone Mass While Cortical Bone Mass and Biomechanical Properties Are Unaltered

**FIGURE 1:**
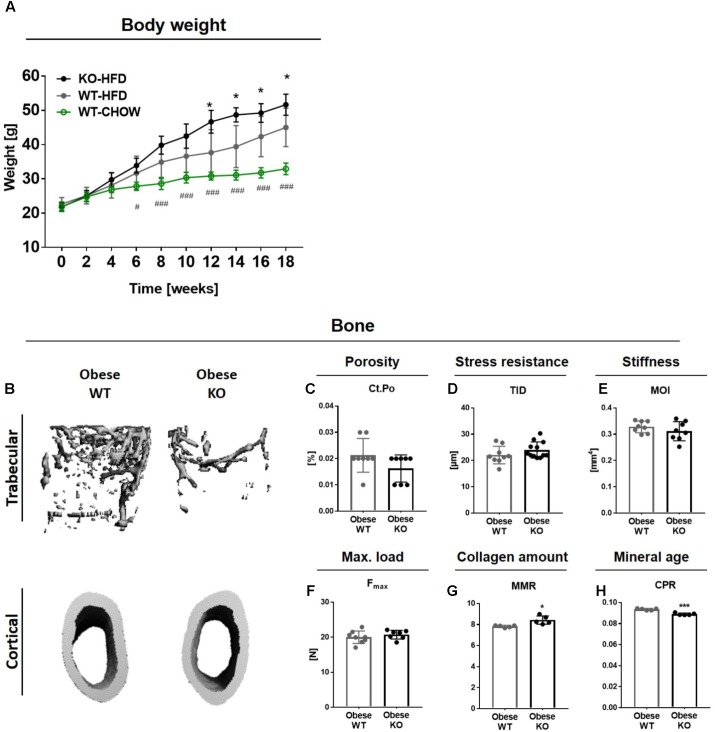
Obese Thy-1^−/−^ mice display decreased trabecular bone mass while cortical bone mass and biomechanical properties are unaltered. Wildtype (WT) and Thy-1^−/−^ (KO) mice were fed with a high fat diet for 18 weeks (HFD). As control group, WT mice were fed a standard chow (CHOW) for the same time period. **(A)** The body weight of standard and HFD fed WT and KO mice over 18 weeks. Hashtags denote significance level of #*P* < 0.05, ###*P* < 0.001 between WT-HFD and WT-CHOW and asterisks denote significance level of ^∗^*P* < 0.05 between WT-HFD and KO-HFD (Student’s *t*-test). **(B)** Representative 3D-images of the trabecular (upper row) and cortical bone compartment (lower row) of the femur. Bone values are presented in **Supplementary Table 2**. **(C)** Cortical porosity (Ct.Po), **(D)** total indentation distance (TID, reference-point indentation), **(E)** moment of inertia (MOI, μCT calculation), and **(F)** maximum force (F_max_, three-point bending test) to fracture cortical bone were examined. **(G)** Mineral-to-matrix ratio (MMR) and **(H)** carbonate-to-phosphate ratio were analyzed using fourier-transform infrared spectroscopy (FTIR). Each point represents one mouse and median ± SD is presented. Asterisks denote significance level of ^∗^*P* < 0.05, ^∗∗∗^*P* < 0.001 (Student’s *t*-test).

Since diet-induced obesity alters bone remodeling leading to decreased femoral trabecular bone mass in mice (Cao et al., 2009, 2010; Halade et al., 2011; Patsch et al., 2011; Picke et al., 2018b), we investigated the impact of Thy-1 on the disturbed bone remodeling in obesity. A significantly higher weight gain was observed beginning 12 weeks after starting a HFD in Thy-1^−/−^ compared to WT mice. As control, one group of WT mice was fed a standard chow diet (CHOW, **Figure 1A**). The impact of Thy-1 deficiency on characteristics of the femoral and tibial long bones as well as of lumbar vertebral bodies after 18 weeks of HFD was analyzed. Similar to mice fed a HFD for 8 weeks (Paine et al., 2018), lack of Thy-1 reduced femoral trabecular bone mass (**Figure 1B** and **Supplementary Animation 1** and **Supplementary Table 2A**) due to a decreased trabecular number and increased trabecular separation (**Supplementary Table 2A**). In addition, we could show that in contrast to lean mice, Thy-1 deficiency also reduced bone mass of the lumbar vertebral bodies under obese conditions (**Supplementary Figure 1** and **Supplementary Table 2D**).

Because HFD-induced obesity often results in an elevated cortical bone mass in humans while fracture risk is highly increased (Cobayashi et al., 2005; Hsu et al., 2006; Zhao et al., 2007; Hardcastle et al., 2015), we analyzed the bone mass and biomechanical properties of the cortical bone compartment. The cortical bone mass and cortical thickness were not altered in obese Thy-1^−/−^ mice (**Figure 1B** and **Supplementary Table 2B**). Cortical porosity was not affected by Thy-1 deficiency after HFD (**Figure 1C**). Using reference-point indentation, we detected no differences between WT and Thy-1^−/−^ mice regarding the total indentation distance (TID, **Figure 1D**), a marker for hardness of cortical bone (Thurner, 2009). In line with that, the moment of inertia (MOI, **Figure 1E**), which is a geometrical parameter that is indicative of resistance to load, and the total tissue area, cortical area, and marrow area (Tt.Ar, Ct.Ar, Ma.Ar, **Supplementary Table 2B**) as well as the femur length and width (**Supplementary Table 2C**) were likewise unaffected by Thy-1 deficiency after HFD. By performing a three-point bending test, we could validate the results of the geometric measurement. The same force was needed to fracture the femora from WT and Thy-1^−/−^ mice (**Figure 1F**). Moreover, in Thy-1^−/−^ mice, the collagen amount within the bone matrix was decreased, indicated by an elevated mineral-to-matrix ratio (MMR, +8%, **Figure 1G**) analyzed by FTIR (Boskey and Pleshko Camacho, 2007). Taking into account the decreased tissue mineral density measured by μCT (**Supplementary Table 2**), the mineral age was younger shown by a reduced carbonate-to-phosphate ratio (CPR, −5%, **Figure 1H**).

In summary, in obesity, lack of Thy-1 resulted in reduced bone mass while bone stiffness was not affected.

### Lack of Thy-1 Reduces Osteoblast Differentiation as Well as Bone Formation and Increases Osteoclast Differentiation and Bone Resorption in Obese Mice

Bone remodeling is a result of the balanced activity of bone-forming osteoblasts and bone-resorbing osteoclasts resulting in a similar bone formation and resorption rate (Crockett et al., 2011). Therefore, both the differentiation and activity of osteoblasts and osteoclasts were analyzed in obese Thy-1^−/−^ and obese WT mice.

**FIGURE 2:**
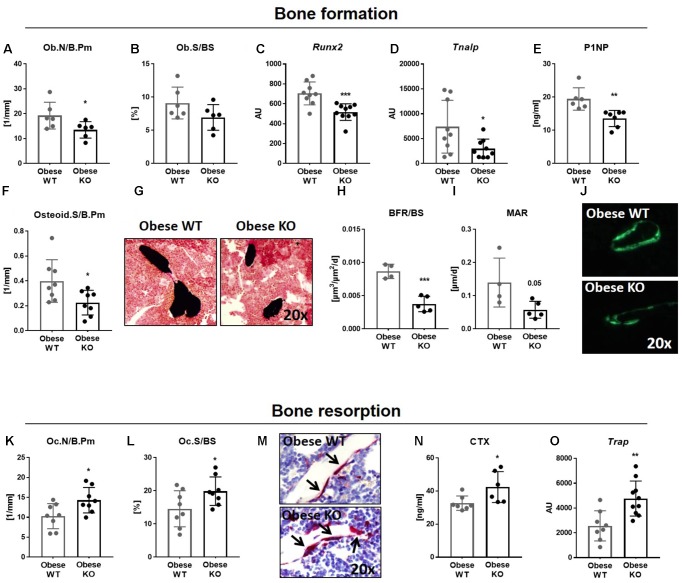
Lack of Thy-1 reduces osteoblast differentiation as well as bone formation and increases osteoclast differentiation and bone resorption in obese mice. Wildtype (WT) and Thy-1^−/−^ (KO) mice were fed with a high fat diet for 18 weeks (HFD). **(A–J)** Bone formation and (K-O) bone resorption were analyzed. The **(A)** osteoblast number per bone perimeter (Ob.N/B.Pm), **(B)** osteoblast surface per bone surface (Ob.S/BS) were analyzed using histology methods. Gene expression of the osteogenic markers **(C)** runt-related transcription factor 2 (*Runx2*), **(D)** and alkaline phosphatase (*Tnalp*) was analyzed by RT-PCR technique. **(E)** The serum concentration of total procollagen type 1 amino-terminal propeptide (P1NP) was analyzed using ELISA technique and the **(F)** osteoid surface per bone perimeter (Osteid.S/B.Pm) by histology. **(G)** Representative sections of von Kossa/van Gieson staining of bone (black) and cartilage (dense red area close to the bone). **(H)** The bone formation rate per bone surface (BFR/BS) as well as **(I)** mineral apposition rate (MAR) were determined by histomorphometric analysis (double calcein labeling). **(J)** Representative images of calcein labeling (green). **(K)** Osteoclast number per bone perimeter (Oc.N/B.Pm) and **(L)** osteoclast surface per bone surface (Oc.S/BS) were analyzed by staining of tartrate resistant acid phosphatase (TRAP). **(M)** Representative images of TRAP staining (red spots and black arrows = TRAP-positive cell/osteoclast). **(N)** Serum concentration of the bone resorption marker carboxy-terminal collagen crosslinks (CTX) was measured by ELISA technique. **(O)** Gene expression of *Trap* was evaluated via RT-PCR. Each point represents one mouse and median ± SD is presented. Asterisks denote significance level of ^∗^*P* < 0.05, ^∗∗^*P* < 0.01, ^∗∗∗^*P* < 0.001 (Student’s *t*-test).

Concerning bone formation, we observed a diminished number, differentiation and activity of osteoblasts (**Figures 2A–J**). The number of osteoblasts (Ob.N/B.Pm, −30%, **Figure 2A**) was decreased in obese Thy-1^−/−^ mice, while osteoblast surface per bone surface (Ob.S/BS, **Figure 2B**) showed trends toward a reduction compared to the WT mice. In accordance, the gene expression of the osteogenic markers *Runx2* (−27%, **Figure 2C**) and *Tnalp* (−60%, **Figure 2D**) were decreased in bones of obese Thy-1^−/−^ mice. Reduced serum concentration of the bone formation marker P1NP (−27%, **Figure 2E**) indicated an attenuated osteoblast activity. Decreased osteoid surface (OS/B.Pm, −43%, **Figures 2F,G**), which indicates impaired mineralization, and decreased bone formation rate (BFR/BS, −57%, **Figures 2H,J**) due to a lower mineral apposition rate (MAR, −59%, **Figures 2I,J**), substantiated these findings. These data were validated in the lumbar vertebral body shown by a reduced bone formation rate and mineral surface per bone surface (BFR/BS, −51%, MS/BS, −46%, **Supplementary Table 2F**).

In parallel to the analysis of osteoblast differentiation and activity in obese Thy-1^−/−^ mice, we examined the impact of Thy-1 on bone resorption. Indeed, osteoclast number (Oc.N/B.Pm, +39%, **Figures 2K,M**) and surface (Oc.S/BS, +36%, **Figures 2L,M**) were increased in the tibia and lumbar vertebral body (**Supplementary Table 2G**) in obese Thy-1^−/−^ mice. Additionally, the concentration of CTX, a serum bone resorption marker, was elevated (+30%, **Figure 2N**). Correspondingly, we observed an elevated gene expression of *Trap*, a marker of osteoclast activity (+86%, **Figure 2O**), in bone of obese Thy-1^−/−^ mice.

### Lack of Thy-1 Promotes Obesity Mediated Inflammation

**FIGURE 3:**
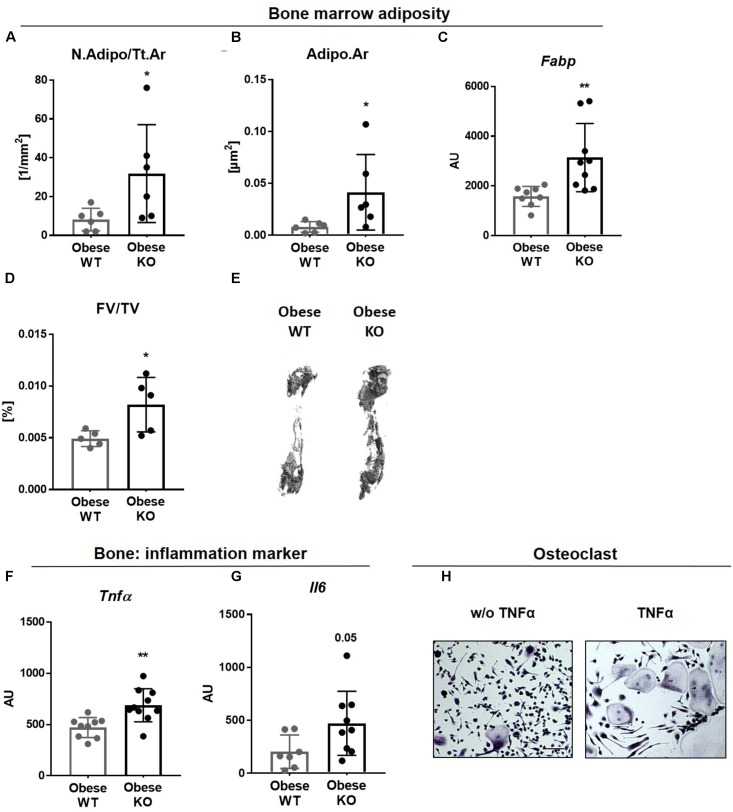
Lack of Thy-1 promotes obesity mediated inflammation. Wildtype (WT) and Thy-1^−/−^ (KO) mice were fed with a high fat diet for 18 weeks (HFD). **(A)** Number of adipocytes per total area (N.Adipo/Tt.Ar) and **(B)** adipocyte area (Adipo.Ar) of adipocytes were analyzed by histology technique. **(C)** Gene expression of the fat marker fatty-acid-binding protein (*Fabp*) in bone was assessed by RT-PCR. **(D)** Fat volume (FV/TV) in the femoral medullary cavity was analyzed by osmium tetroxide staining. **(E)** Representative 3D-images of the fat volume of whole femur. **(F,G)** Gene expression of the pro-inflammatory markers tumor necrosis factor α (*Tnfα*) and interleukin 6 (*Il6*). **(H)** Osteoclast precursor cells from WT mice were cultured *ex vivo* without (w/o) and with TNFα and osteoclastogenesis was detected via staining for tartrate resistant acid phosphatase (TRAP; giant, multinucleated, red cells = osteoclasts; indicated by arrows). Asterisks denote significance level of ^∗^*P* < 0.05, ^∗∗^*P* < 0.01 (Student’s *t*-test).

There is accumulating evidence that excessive adipose tissue accumulation in obesity is detrimental to bone health. Indeed, Thy-1^−/−^ mice gained more weight compared to controls after HFD (Woeller et al., 2015; Paine et al., 2018; Picke et al., 2018a; **Figure 1A**). In addition to an increased adipocyte number (N.Adipo, +2.5-fold, **Figure 3A**), we observed an elevated adipocyte area (Adipo.Ar, +4-fold%, **Figure 3B**) as well as an upregulated gene expression of the fat marker *Fabp* in bone of obese Thy-1^−/−^ mice (+1-fold, **Figure 3C**). Altogether an increased total fat volume (FV/TV, +56%, **Figures 3D,E**) was observed indicating expanded bone marrow adiposity in Thy-1^−/−^ mice. Increased body fat mass is associated with latent inflammatory response (Greco et al., 2015). Consistent with this, the gene expression of the potent pro-inflammatory cytokines *Tnfα* and *Il6* (+130 and +46%, respectively, **Figures 3F,G**) were elevated in obese Thy-1^−/−^ mice. Indeed, TNFα strongly increased osteoclast differentiation shown by an increased number of multinucleated, TRAP positive giant cells (**Figure 3H**).

### Thy-1^−/−^ in Obesity Does Not Alter the Wnt and YAZ/TAZ Pathway

**FIGURE 4:**
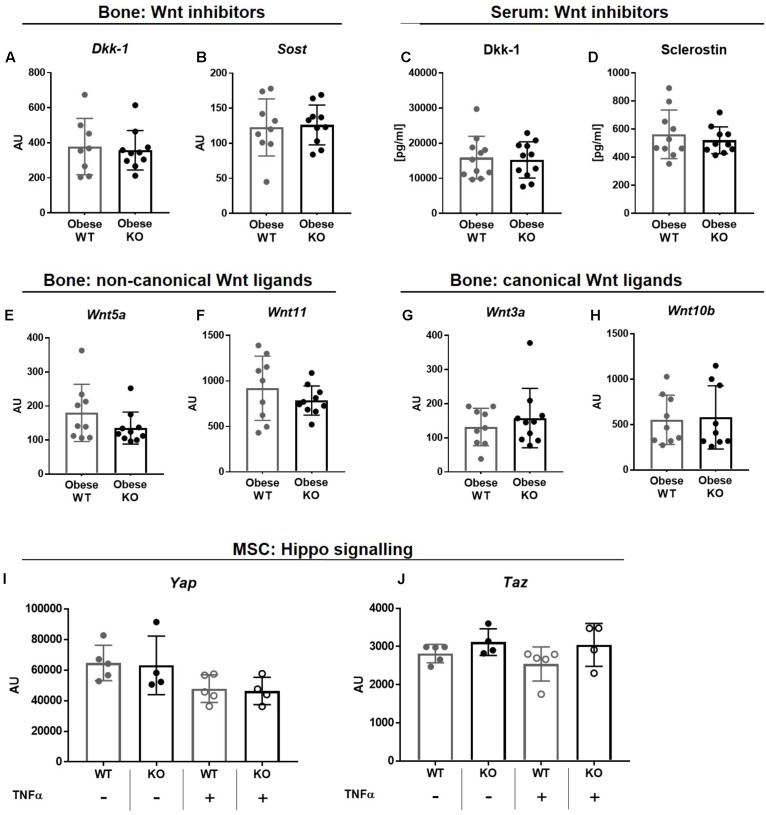
Thy-1^−/−^ in obesity does not alter the Wnt and YAZ/TAZ pathway. Wildtype (WT) and Thy-1^−/−^ (KO) mice were fed with a high fat diet for a time period of 18 weeks (HFD). Gene expression of the Wnt pathway inhibitors **(A)** dickkopf-1 (*Dkk-1*) and **(B)** sclerostin (*Sost*) and **(C,D)** their serum concentrations were evaluated by RT-PCR and ELISA technique, respectively. Gene expression of Wnt ligands such as **(E)** *Wnt5a*, **(F)** *11*, **(G)** *3a*, and **(H)** *10b* in bone was analyzed by RT-PCR. MSCs from WT and Thy-1^−/−^ mice were treated with TNFα and expression of *Yap* and *Taz* of the hippo signaling were investigated. Statistical analysis was performed by **(A–H)** Student’s *t*-test and by **(I,J)** 2-way ANOVA.

Osteogenic differentiation and bone formation is strongly regulated by Wnt signaling that is controlled by inhibitor molecules such as Dkk-1 and sclerostin (Pinzone et al., 2009; Kim et al., 2013). However, gene expression of *Dkk-1* and sclerostin (*Sost*, **Figures 4A,B**) as well as their serum concentrations (**Figures 4C,D**) were unaffected in obese Thy-1^−/−^ mice. In line with these observations, the gene expression of non-canonical, *Wnt5a* and *Wnt11*, as well as canonical Wnt ligands, *Wnt3a* and *Wnt10b*, were not altered in bone by Thy-1 deficiency after HFD (**Figures 4E–H**). The Hippo signaling increases osteoblastogenesis (Pan et al., 2018). Neither Thy-1 deficiency nor treatment with TNFα altered gene expression of YAP or TAZ in wildtype and Thy-1^−/−^ MSCs (**Figures 4I,J**).

In summary, Thy-1 does not alter bone formation by modulation of the Wnt pathway nor the Hippo signaling in Thy-1^−/−^ mice.

### Lack of Thy-1 in Obese Mice Alters the Gene Expression of RANKL, OPG, and CSF1 Under Inflammatory Conditions

**FIGURE 5:**
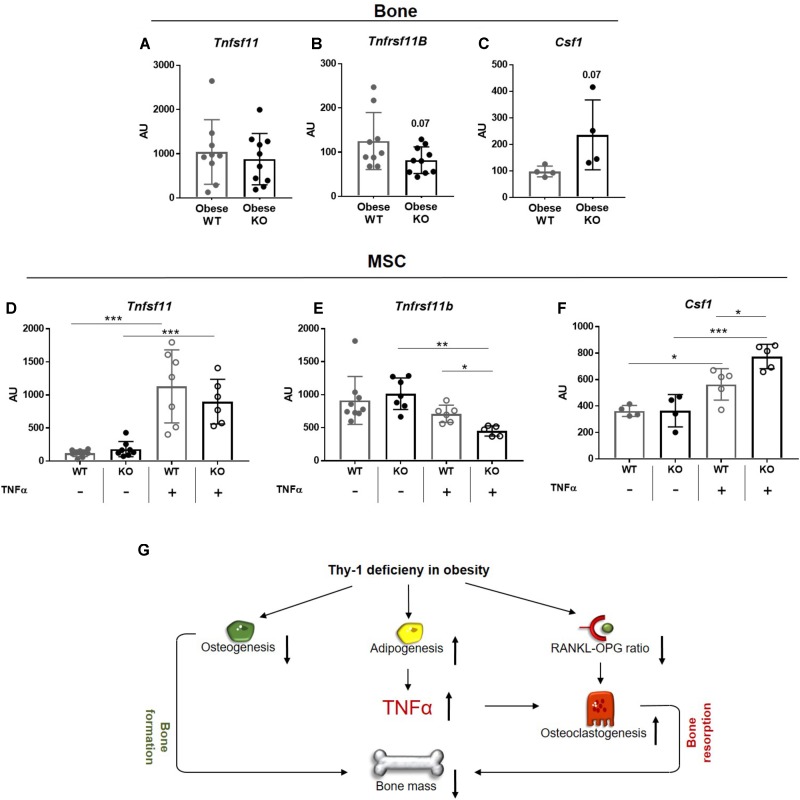
Lack of Thy-1 in obese mice alters the gene expression of RANKL, OPG, and CSF1 under inflammatory conditions. Wildtype (WT) and Thy-1^−/−^ (KO) mice were fed with a high fat diet for a time period of 18 weeks (HFD). **(A)** Gene expression of receptor activator of NF-κB ligand (RANKL, *Tnfsf11*), **(B)** its decoy receptor osteoprotegerin (OPG, *Tnfrsf11b*), and **(C)** receptor of Csf1 *(Cfs1r)* was analyzed in bone. MSCs from WT and KO mice were treated with TNFα for 24 h to mimic an inflammatory environment and gene expression of *Tnfsf11*, *Tnfrsf11b*, and *Cfs1* was determined **(D–F)**. **(G)** Summary figure of the key findings. In mice, Thy-1 deficiency results in a reduced osteoclastogenesis and increased adipogenesis leading to a decreased bone formation. Adipocytes produce more of the pro-inflammatory cytokine TNFα and the RANKL-OPG ratio is reduced resulting in an elevated osteoclastogenesis and poor bone mass. Each point represents one mouse and median ± SD is presented. Asterisks denote significance level of ^∗^*P* < 0.05, ^∗∗^*P* < 0.01, ^∗∗∗^*P* < 0.001 measured by **(A–C)** Student’s *t*-test and by **(D–F)** 2-way ANOVA.

Since the RANK-RANKL-OPG axis and binding of CSF1 to its receptor CSF1R, located at surface of osteoclasts, play central roles in osteoclastogenesis, we analyzed their gene expression in bone of WT and Thy-1^−/−^ mice. The gene expression of *Tnfsf11*, which encodes for RANKL, was unaffected, while the expression of its decoy receptor *Tnfrsf11b*, encodes for OPG (*p* = 0.07) showed trend toward reduction in bone of obese Thy-1^−/−^ mice (**Figures 5A,B**). The expression of *Csf1* was not detectable in bone, but the expression of its receptor *Csf1r* showed a trend toward upregulation (*p* = 0.07, **Figure 5C**). Obesity is characterized by latent inflammation shown by increased TNFα levels. Since RANKL, OPG, and CSF1 are expressed by stromal cells in bone, we stimulated WT and Thy-1^−/−^ MSCs with TNFα to mimic the pro-inflammatory environment. Gene expression analysis revealed that Thy-1 does not affect the expression of *Tnfsf11*, *Tnfrsf11b*, and *Csf1* under basal conditions (**Figures 5D–F**). Upon pro-inflammatory stimulation, Thy-1^−/−^ MSCs expressed reduced levels of *Tnfrs11b* (−36%, **Figure 5E**) while *Csf1* (+37%, **Figure 5F**) expression was elevated.

Taken together, Thy-1 leads to an unaltered *Tnfsf11*, reduced *Tnfrsf11b*, and increased *Csf1* expression in an inflammatory environment that might contribute to increased osteoclastogenesis in Thy-1^−/−^ mice.

## Discussion

Alterations in bone mass and quality caused by osteoporosis and obesity are major health problems worldwide. They are caused by an unbalanced differentiation capacity and activity of bone-forming osteoblasts and bone-resorbing osteoclasts. The underlying mechanisms are still not fully understood and therefore sufficient therapy options are limited. One step toward identifying these mechanisms was the recent discovery that the cell-surface protein Thy-1 is a major modulator of MSC differentiation into the adipogenic or osteogenic lineage (Hosoya et al., 2012; Chung et al., 2013; Picke et al., 2018a). In lean mice, Thy-1 deficiency increases whole body adipogenesis while decreasing osteoblast differentiation resulting in poor bone mass and quality indicating Thy-1 as a protector of bone mass (Woeller et al., 2015; Picke et al., 2018a). Therein, the activity of osteoclasts was not altered. Here, we went one step further and analyzed Thy-1 effects on bone under obese conditions. Thy-1^−/−^ and WT mice fed a HFD developed an obese phenotype whereas Thy-1 deficiency exacerbated fat accumulation. Most importantly, Thy-1 deficiency decreased the bone mass under obese conditions by affecting both bone formation and bone resorption.

Thy-1 has been shown to promote osteogenesis and thereby inhibit adipogenesis (Hosoya et al., 2012; Chung et al., 2013; Woeller et al., 2015; Picke et al., 2018a). More specifically, inhibition of Thy-1 expression in MSCs isolated from different sources resulted in increased adipogenic differentiation mirrored by enhanced lipid droplet accumulation (Moraes et al., 2016). Moreover, ectopic expression of Thy-1 in adipocyte-like 3T3 cells inhibits adipogenic differentiation while depletion of endogenous Thy-1 in human fibroblasts increases their ability to undergo adipogenesis (Woeller et al., 2015; Picke et al., 2018a). Correspondingly, Thy-1^−/−^ mice show an increased weight gain and body fat mass caused by inhibiting the activity of the Fyn kinase resulting in a reduced expression of PPARγ (Woeller et al., 2015; Picke et al., 2018a). On the other hand, Thy-1-positive MSCs, adipose-derived stromal cells and dental pulp cells show an increased ALP activity and mineralization capacity *in vitro* (Hosoya et al., 2012; Chung et al., 2013; Picke et al., 2018a). Upon subcutaneous injection, these cells increase formation of bone-like matrix and improve the healing of critical size defects (Hosoya et al., 2012; Chung et al., 2013). In contrast, Moraes et al. (2016) demonstrated that Thy-1 downregulation supports osteogenic differentiation of dental pulp cells and MSCs but, however, detected concurrently an increased adipogenic differentiation of cells with decreased Thy-1 expression. Importantly, we recently demonstrated that Thy-1^−/−^ mice have a massive reduction of bone mass and quality independent of the gender when fed a standard chow (Picke et al., 2018a).

In the present study, we demonstrated that Thy-1 deficiency augmented obesity-mediated bone loss. In agreement with Paine et al. (2018) we found a reduced femoral bone volume and microstructure due to a decreased trabecular number and increased trabecular separation. Interestingly, bone mass of vertebral bodies was not altered in lean Thy-1^−/−^ mice, but reduced in obese Thy-1^−/−^ mice. This is an interesting finding as other studies also report varying effects on femur and vertebral bodies in rodent experiments. The negative effect of osteoporosis induced by ovariectomy on bone mass was found to be more pronounced in femur neck compared to the spine (Jiang et al., 1997). One reason could be that the spine of quadrupeds is in a horizontal position and could be therefore exposed to less mechanical loading in comparison to the femur (Smit, 2002). The increased mechanical loading brought about by weight gain in lean Thy-1^−/−^ mice may not have been sufficient to affect trabecular bone mass at sites other than the femur.

In lean Thy-1^−/−^ mice, the cortical bone mass and its biomechanical properties were significantly reduced compared to WT controls (Picke et al., 2018a). In contrast, HFD and Thy-1 deficiency did not alter the cortical bone mass. Consequently, bone quality markers such as cortical porosity and the moment of inertia were not affected by Thy-1 deficiency under obese conditions. Thy-1-mediated effects on cortical bone therefore seem to be compensated by as yet unknown mechanisms in obesity. In addition, in lean mice, Thy-1 deficiency was accompanied by increased expression of Wnt inhibitors, reduced expression of Wnt ligands and reduced responsiveness to Wnt stimulation (Picke et al., 2018a). However, obesity seems to overwrite these Thy-1-mediated effects on the Wnt pathway. Further, osteo-anabolic Hippo signaling was also not altered by neither Thy-1 deficiency nor mimicked the pro-inflammatory environment *ex vivo*.

Bone remodeling is a result of the balanced bone formation by bone-forming osteoblasts and bone resorption by bone-resorbing osteoclasts (Crockett et al., 2011). As shown in lean and obese mice Thy-1 deficiency reduced the number and activity of osteoblasts and thus impaired bone formation (Paine et al., 2018; Picke et al., 2018a). Interestingly, in lean mice, lack of Thy-1 neither affected number nor activity of osteoclasts. In contrast, upon 18 weeks of HFD Thy-1 deficiency enhanced the number and activity of osteoclasts and, thus, might force bone resorption. This might contribute to bone loss in obese Thy-1 deficient mice. Interestingly, Paine et al. (2018) described a reduction of osteoblasts in obese Thy-1^−/−^ mice while osteoclasts were unaffected. However, Paine et al. (2018) analyzed mice upon 8 weeks of HFD. In our study, Thy-1 deficiency induced an enhanced weight gain from 12 weeks of HFD. Thus, Thy-1 deficiency impaired osteoblast differentiation and activity under both lean (Picke et al., 2018a) body composition and in the early and late phase of obesity. In contrast, an impact of Thy-1 deficiency on osteoclasts was only seen in late, distinct obesity.

Obesity is associated with latent inflammatory responses indicated by an elevated expression of pro-inflammatory cytokines such as Il1, Il6, and TNFα (Cao, 2011). Indeed, we detected an increased bone marrow adiposity in obese Thy-1^−/−^ mice. Correspondingly, gene expression of *Il6* and *Tnfα* was up-regulated in the bone of obese Thy-1^−/−^ compared to obese WT mice. IL-6 can be produced by osteoblasts and induces bone resorption (Ishimi et al., 1990). TNFα expression has been shown to be increased in obesity, arthritis, and osteoporosis. TNFα is able to affect both osteoclast differentiation and osteogenesis. TNFα produced by adipocytes potently reduces the differentiation of osteoblasts via TNFR1 or via upregulation of autophagy and reduction of apoptosis (Gilbert et al., 2000, 2005; Zhao et al., 2011; Abuna et al., 2016; Zheng et al., 2017). In obesity, arthritis, and osteoporosis, which are diseases that are characterized by a reduction of bone mass due to an increased osteoclastogenesis (Cao, 2011; Halade et al., 2011; Shu et al., 2015). TNFα has the ability to increase osteoclast differentiation by elevating the expression of RANKL, a master modulator of osteoclastogenesis, by osteoblasts via TNFR1 and/or by the Pi3K/Akt pathway (Zhang et al., 2001; Siggelkow et al., 2003; Kitaura et al., 2013; Osta et al., 2014; Wu et al., 2017). TNFα alone does not increase the differentiation of osteoclast precursor cells (Kobayashi et al., 2000; Lam et al., 2000), but has been shown to act synergistically together with RANKL on osteoclastogenesis (Azuma et al., 2000; Lam et al., 2000; Fuller et al., 2002). In addition, TNFα can also elevate osteoclast differentiation when CSF1 is present (Kobayashi et al., 2000; Kitaura et al., 2005). *Ex vitro*, we demonstrated the augmentation of osteoclastogenesis by TNFα in the presence of RANKL and CSF1. Thus, despite of similar expression of *Tnfsf11* (RANKL) in bone of obese WT and Thy-1-deficient mice elevated amounts of TNFα in Thy-1-deficient mice might contribute via the synergistic action with RANKL to increased osteoclast number and activity in Thy-1 deficient mice.

In addition to the RANK-RANKL-OPG axis, the presence of CSF1 is essential for osteoclast differentiation (Kim and Kim, 2016). The reduced expression of RANKL decoy receptor, *Tnfsf11b* (OPG) and increased expression of *Csf1* under pro-inflammatory conditions in Thy-1^−/−^ MSC might contribute to the increased osteoclastogenesis in obese Thy-1^−/−^ mice.

However, one limitation of our study is that we could not demonstrate a direct relation *in vivo* between alteration of TNFα, CSF1, and OPG expression in obese Thy-1^−/−^ mice and increased osteoclastogenesis and diminished bone mass. Because we were working with a global knockout mouse model, we cannot fully exclude effects of other cell types expressing Thy-1 on bone metabolism. Nevertheless, we showed previously that isolated MSCs from lean Thy-1^−/−^ mice created less mineralized matrix in a WT environment *in vivo* (Picke et al., 2018a). However, we failed to discover the underlying mechanism of reduced osteoblastogenesis in Thy-1 deficiency in obese mice. It is known that obese patients have an increased concentration of unsaturated fatty acids that negatively affect osteoblastogenesis (Hardouin et al., 2016). In future, it should be addressed if this could also affect differentiation and function of Thy-1^−/−^ MSCs.

Taken together, Thy-1 controls the balance between bone formation and bone resorption. In lean mice and in the early phase of obesity, a lack of Thy-1 impairs bone formation by inhibition of osteoblast differentiation, while there is no effect on bone resorption (Paine et al., 2018; Picke et al., 2018a). In the present study, we show that a lack of Thy-1 under manifest obese conditions affects both bone formation and bone resorption. Obese Thy-1^−/−^ mice exhibited increased bone marrow adiposity associated with an increased pro-inflammatory environment including increased *Tnfa* expression. TNFα is a strong promotor of osteoclast differentiation and, thus, bone resorption. On the other hand lack of Thy-1 resulted in an attenuated expression of *Tnfsf11B* (OPG), the decoy receptor for *Tnfsf11* (RANKL). Both increased TNFα expression and diminished *Tnfsf11B* might induce bone resorption in obese Thy-1^−/−^ mice (**Figure 5G**).

## Author Contributions

A-KP and AS designed the study, performed the experiments, analyzed and interpreted the data, and wrote the manuscript. UA provided the experiences and methods for RNA analysis. MR and LH provided the skeletal expertise, methods for bone analysis, and interpreted the bone data. GC performed part of the μCT measurements and analyzed the data. FS and BB carried out the compositional analyses. JS discussed the data and revised the manuscript. All authors discussed the data and read and edited the manuscript.

## Conflict of Interest Statement

The authors declare that the research was conducted in the absence of any commercial or financial relationships that could be construed as a potential conflict of interest.
