# Supplementary material for: Thy-1 Deficiency Augments Bone Loss in Obesity by Affecting Bone Formation and Resorption
Source: Front Cell Dev Biol. 2018 Oct 2;6:127. doi: 10.3389/fcell.2018.00127 (PMC6176687; doi:10.3389/fcell.2018.00127)
Supplement: Supplementary file 1 [file Data_Sheet_1.docx]

Supplementary Material

Thy-1 deficiency augments bone loss in obesity by affecting bone formation and resorption

Ann-Kristin Picke^*^, Graeme M. Campbell, Felix N. Schmidt, Björn Busse, Martina Rauner, Ulf Anderegg, Lorenz C. Hofbauer and Anja Saalbach

*** Correspondence:**

Dr. Ann-Kristin Picke
[ann-kristin.picke@uni-ulm.de](mailto:ann-kristin.picke@uni-ulm.de)

# Supplementary Figure 1


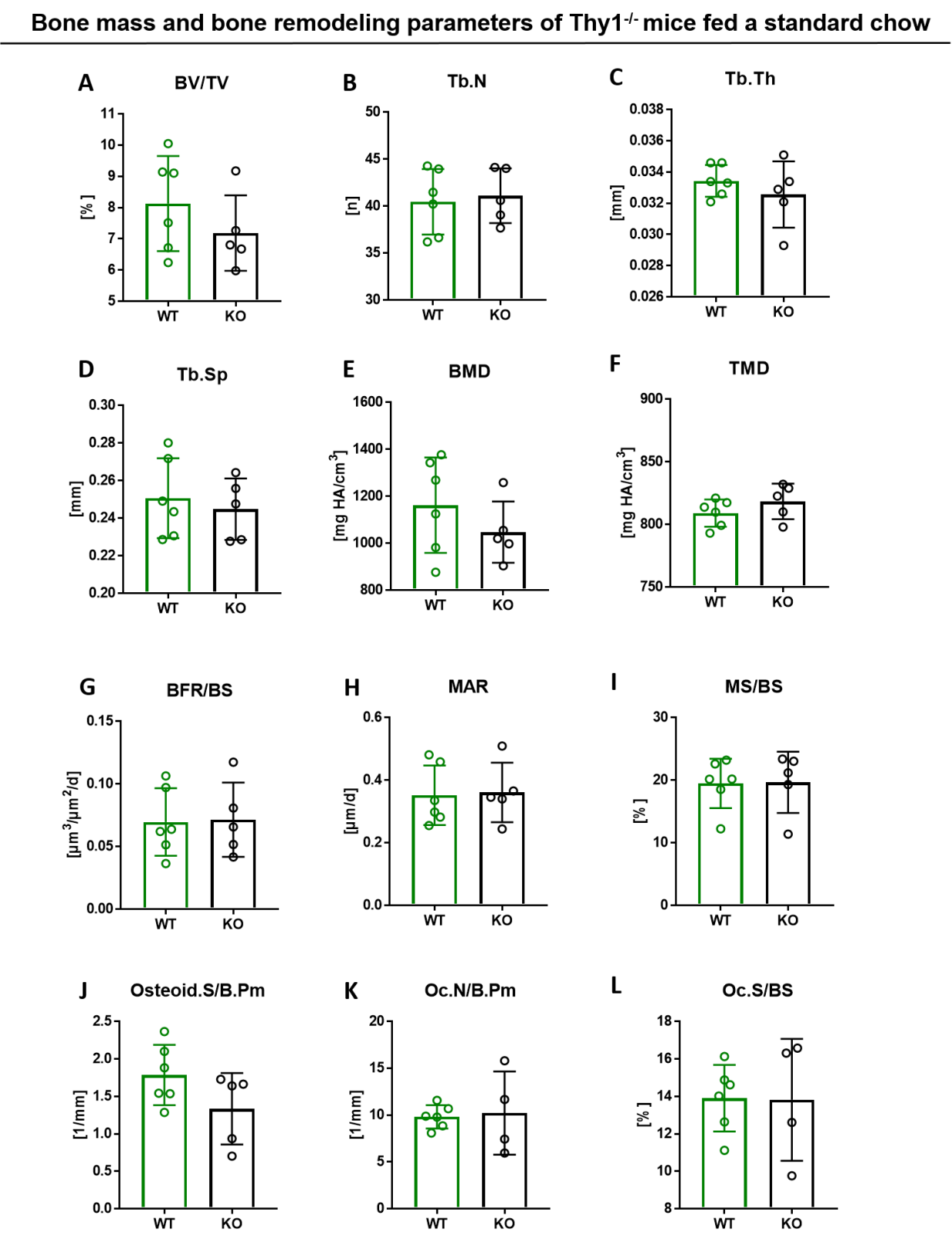


**Thy-1^-/-^ does not affect bone mass and remodelling of vertebral body after chow diet**. Wildtype (WT) and Thy-1^-/-^ (KO) mice were fed a standard chow. At the age of 12 weeks, A) the trabecular bone volume per total volume (BV/TV), B) trabecular number (Tb.N), C) thickness (Tb.Th) and D) separation (Tb.Sp), E) the bone mineral density (BMD) and F) total mineral density (TMD) of the 4^th^ lumbar vertebral body were analysed via µCT technique. On the histological level, the G) bone formation rate per bone surface (BFR/BS), H) mineral apposition rate (MAR), and I) mineral surface per bone surface (MS/BS) were determined by double calcein labelling, J) the osteoid surface per bone perimeter (Osteoid.S/B.Pm) by von Kossa/Safranin-O staining, and the K) osteoclast number per bone perimeter (Oc.N/B.Pm) and L) osteoclast surface per bone surface (Oc.S/BS) by TRAP staining. Each point represents one mouse and median ± standard deviation are presented.

# Supplementary Table 1: Murine primer sequences for real-time qPCR

| **Target gene** | **Primer sequences 5’ – 3’** | |
| --- | --- | --- |
|  | **Sense** | **antisense** |
| *Fabp* | ATGTGTGATGCCTTTGTGGGAAC | TCATGTTGGGCTTGGCCATG |
| *Il6* | GCCTATTGAAAATTTCCTCTGG | AAGATGAATTGGATGGTCTTGG |
| *Tnfα* | CTTAGACTTTGCGGAGTCCG | ACAGTCCAGGTCACTGTCCC |
| *Dkk-1* | TCACACCAAAGGACAAGAAGG | GTAACAGCGCTGGAATATCTCC |
| *Sost* | CTCACCCGCTTCCACAAC | GGCAGAGTCTGGGACTCAAG |
| *Runx2* | CTCCAAGACCCTAAGAAACCG | TCTCTCAGATACCATGGGTGC |
| *Tnalp* | TGTGGTTACTGCTGATCATTC | TTGTGAGCGTAATCTACCATGG |
| *Trap* | ACTTGCGACCATTGTTAGCC | AGAGGGATCCATGAAGTTGC |
| *Tnfsf11* | GCAGATTTGCAGGACTCGAC | TATGGGAACCCGATGGGATG |
| *Tnfrsf11b* | AGTTTGCCTGGGACCAAAGTG | CTGTGGTGAGGTTCGAGTGG |
| *Gapdh* | TGGCAAAGTGGAGATTGTTGCC | AAGATGGTGATGGGCTTCCCG |
| *Thy-1* | TCCATCCAGCATGAGTTC | TGAACCAGCAGGCTTATG |
| *Rs36* | GGACCCGAGAAGACCTCCTT | GCACATCACTCAGAATTTCAATGG |
| *YAP* | TCAATCCCAGCACAGCAAATG | CGAGAGTGATAGGTGCCACTG |
| *TAZ* | CTTGGGCAAATGTGTGCCTG | TTTTCTGTCCAAAGCGGGGG |
| *Csf1* | CAACACCCCCAATGCTAACG | CCACATGGCCTCGGCTAGAG |
| *Csf1r* | TGACCCTACTCAGTTGCCCT | TCAGGGCCTCCTTCTCATCAG |
| *Wnt3a* | GTGCGACCTGTTGTGCTG | CCAGACAGGAGCTTCTCCC |
| *Wnt5a* | ACATTGGAGAAGGTGCGAAG | GTCTCTCGGCTGCCTATTTG |
| *Wnt10b* | TCTCTTTCAGCCCTTTGCTCGGAT | ACAACTGAACGGAAGGAGAAGCCT |
| *Wnt11* | TGCTGCTACGTCACCTGC | AGTGGAGGCACAGCACAAG |
| *Wnt16* | GGAGCTGTGCAAGAGGAAAC | AGTGGCGACCATACAGTTCC |

Fabp = fatty-acid-binding protein, Il6 = interleukin 6, Tnfα = tumor necrosis factor α, *Dkk-1* = dickkopf 1 =, *Sost* =sclerostin, *Runx2* = runt-related transcription factor 2, *Tnalp* = alkaline phosphatase, *Trap* = tratrate restistant acid phosphatase, *Rankl* = receptor activator of NF-κB ligand, *Gapdh* = glyceraldehyde 3-phosphate dehydrogenase, *RS36 =* ribosomal protein, large, P0 (Rplp0), *Yap* = yes-associated protein, *Taz* = PDZ-binding motif, *Csf1* = colony stimulating factor 1, *Csf1r* = receptor of Csf1, *Wnt* = Wnt ligand

**Supplemetary Table 2:** Bone mass and bone remodelling parameter of obese WT and Thy-1^-/-^ mice

| **Parameter (unit)** | **WT HFD** | **KO HFD** | **P-value** | **WT CHOW vs. KO CHOW** |
| --- | --- | --- | --- | --- |
| **BONE** | | | | |
| **A. Femur - trabecular bone compartment (µCT analysis)** | | | | |
| BV/TV (%) | 6.54 ± 0.94 | 4.06 ± 1.47 | <0.001 | <0.05 |
| Tb.N (n) | 29.9 ± 4.23 | 23.4 ± 0.22 | <0.05 | ns |
| Tb.Th (mm) | 0.060 ± 0.003 | 0.056 ± 0.001 | ns | <0.05 |
| Tb.Sp (mm) | 0.34 ± 0.06 | 0.48 ± 0.01 | <0.05 | ns |
| **B. Femur - cortical bone compartment (µCT analysis)** | | | | |
| BV/TV (%) | 93.0 ± 0.62 | 92.5 ± 0.21 | ns | <0.01 |
| Ct.Th (µm) | 0.03 ± 0.002 | 0.02 ± 0.001 | ns | <0.01 |
| BMD (mg HA/cm^3^) | 8,919 ± 483 | 8,981 ± 158 | ns | <0.01 |
| TMD (mg HA/cm^3^) | 11,230 ± 177 | 11,185 ± 151 | ns | <0.01 |
| Tt.Ar (mm^2^) | 1.83 ± 0.06 | 1.76 ± 0.11 | ns | ns |
| Ct.Ar (mm^2^) | 0.71 ± 0.03 | 0.71 ± 0.03 | ns | ns |
| Ma.Ar (mm^2^) | 1.05 ± 0.06 | 0.99 ± 0.08 | ns | ns |
| **C. Total Femur (caliper analysis)** | | | | |
| Length (mm) | 15.7 ± 0.21 | 15.4 ± 0.27 | ns | <0.05 |
| Width (mm) | 1.56 ± 0.03 | 1.58 ± 0.09 | ns | <0.01 |
| **D. Vertebral body - trabecular bone compartment (µCT analysis)** | | | | |
| BV/TV (%) | 11.0 ± 1.30 | 8.41 ± 2.57 | <0.05 | ns |
| Tb.N (n) | 45.6 ± 2.19 | 41.2 ± 1.49 | <0.01 | ns |
| Tb.Th (µm) | 0.037 ± 0.003 | 0.036 ± 0.002 | ns | ns |
| Th.Sp (µm) | 0.22 ± 0.010 | 0.24 ±0.009 | <0.01 | ns |
| BMD (mg HA/cm^3^) | 1433 ± 191 | 1238 ± 197 | <0.05 | ns |
| TMD (mg HA/cm^3^) | 841 ± 21.5 | 842 ± 7.66 | ns | ns |
| **BONE FORMATION** | | | | |
| **E. Tibia -** **trabecular bone compartment (histological analysis)** | | | | |
| BFR/BS (µm^3^/µm^2^/d) | 0.009 ± 0.001 | 0.004 ± 0.001 | <0.001 | =0.05 |
| MAR (µm/d) | 0.14 ± 0.07 | 0.06 ± 0.03 | =0.05 | ns |
| MS/BS (%) | 7.25 ± 2.88 | 6.88 ± 1.54 | ns | <0.05 |
| Osteoid.S/B.Pm (1/mm) | 0.40 ± 0.17 | 0.23 ± 0.01 | <0.01 | <0.01 |
| **F. Vertebral body -** **trabecular bone compartment (histological analysis)** | | | | |
| BFR/BS (µm^3^/µm^2^/d) | 0.014 ± 0.003 | 0.007 ± 0.001 | <0.01 | ns |
| MAR (µm/d) | 0.13 ± 0.04 | 0.11 ± 0.02 | ns | ns |
| MS/BS (%) | 11.2 ±1.94 | 6.10 ± 0.91 | <0.01 | ns |
| Osteoid.S/B.Pm (1/mm) | 0.57 ± 0.13 | 0.50 ± 0.25 | ns | ns |
| **Serum** | | | | |
| P1NP | 19.5 ± 3.37 | 14.3 ± 1.74 | <0.01 | <0.05 |
| **BONE RESORPTION** | | | | |
| **G. Tibia -** **trabecular bone compartment (histological analysis)** | | | | |
| Oc.N/B.Pm (1/mm) | 9.16 ± 2.02 | 11.8 ± 1.36 | <0.01 | ns |
| Oc.S/BS (%) | 13.3 ± 3.00 | 23.3 ± 3.93 | <0.001 | ns |
| **H.** **Vertebral body -** **trabecular bone compartment (histological analysis)** | | | | |
| Oc.N/B.Pm (1/mm) | 9.16 ± 2.02 | 11.8 ± 1.36 | <0.01 | ns |
| Oc.S/BS (%) | 13.3 ± 2.99 | 23.3 ± 3.93 | <0.001 | ns |
| **Serum** | | | | |
| CTX | 32.7 ± 4.34 | 42.4 ± 9.37 | <0.05 | ns |
| **GENERAL** | | | | |
| Body weight | 44.7 ± 5.98 | 51.6 ± 3.08 | <0.05 | <0.01 |

Wildtype (WT) and Thy-1^-/-^ (KO) mice were fed with a high fat diet (HFD) for 18 weeks. In the right column the obtained data from lean WT and Thy-1^-/-^ mice after standard lab diet (CHOW) were compared to each other to underline differences between WT and Thy-1^-/-^ mice that were fed with CHOW or HFD (cohort from Picke et al. Sci Transl Med. 2018 Aug 8;10(453). pii: eaao6806.). BV/TV = bone volume per total volume, Tb.N = trabecular number, Tb.Th = trabecular thickness, Tb.Sp = trabecular separation, BMD = bone mineral density, TMD = tissue mineral density, HA = hydroxyapatite, Ct.Th = cortical thickness, Tt.Ar = total area, Ct.Ar = cortical area, Ma.Ar = marrow area, MS/BS = mineral surface per bone surface, BFR/BS = bone formation rate per bone surface, MAR = mineral apposition rate, MS/BS = mineral surface per bone surface, P1NP = procollagen type 1 amino-terminal propeptide, Oc.N/B.Pm = osteoclast number per bone perimeter, Oc.S/BS = osteoclast surface per bone surface, CTX = carboxy-terminal collagen crosslinks
