# Supplementary material for: Thy-1 Deficiency Augments Bone Loss in Obesity by Affecting Bone Formation and Resorption
Source: Front Cell Dev Biol. 2018 Oct 2;6:127. doi: 10.3389/fcell.2018.00127 (PMC6176687; doi:10.3389/fcell.2018.00127)
Supplement: Supplementary file 2 [file Presentation_1.PPTX]

## Slide 1
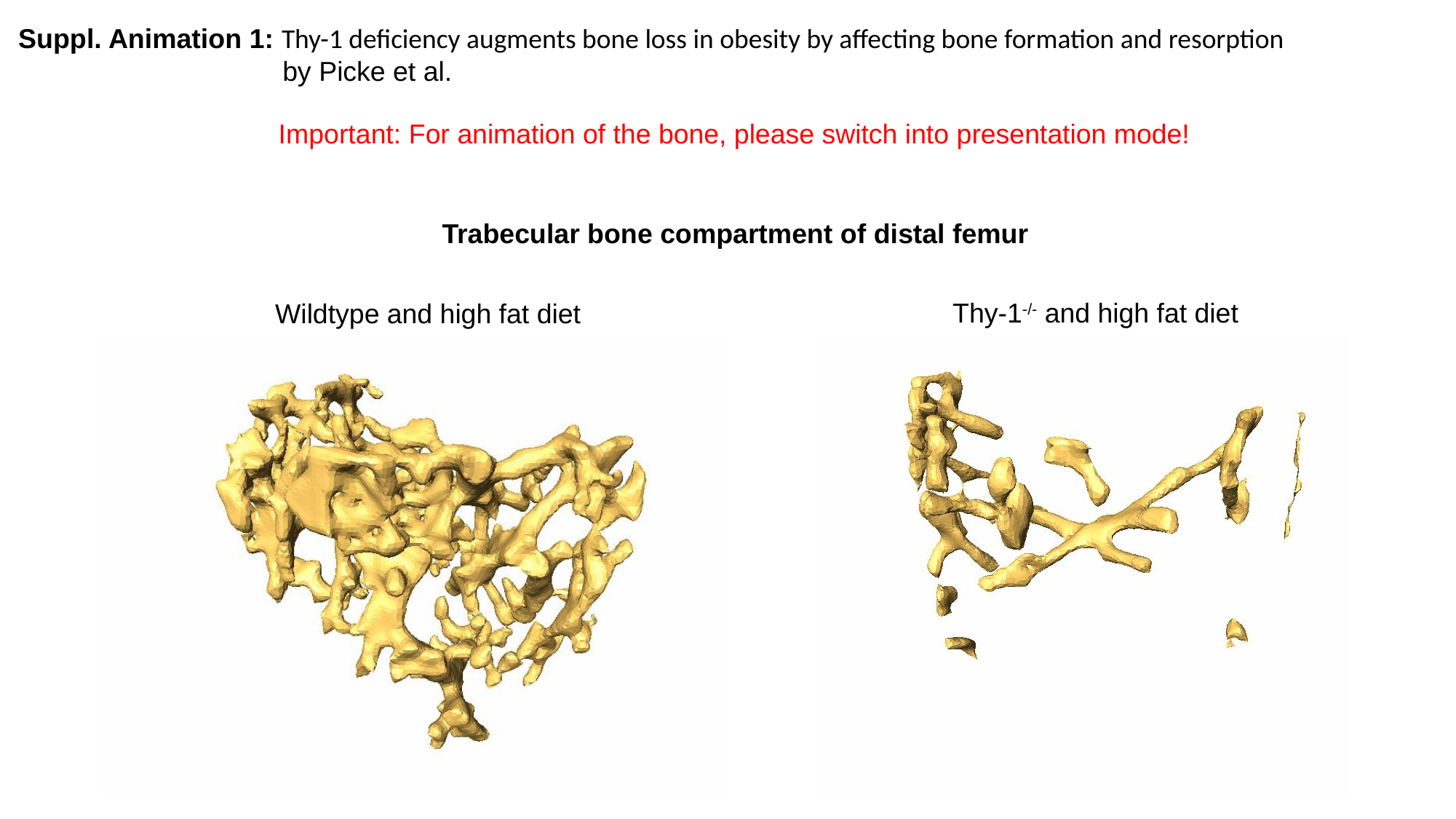

Suppl. Animation 1: Thy-1 deficiency augments bone loss in obesity by affecting bone formation and resorption
		 by Picke et al.
Important: For animation of the bone, please switch into presentation mode!
Trabecular bone compartment of distal femur
Thy-1-/- and high fat diet
Wildtype and high fat diet
